# Bullets in the Brain and the Roentgen Rays

**Published:** 1897-04

**Authors:** 


### BULLETS IN THE BRAIN AND THE ROENTGEN RAYS.

A. Eulenburg (*Deutschen Med. Woch.*, August 17, 1896,) relates two cases in which it was possible to localize bullets in the intracranial cavity by means of radiography.

CASE I. A man, aged eighteen, accidentally shot himself in the head with a revolver. The bullet entered 3½ cm. above and 2 cm. in front of the attachment of the ear. There was complete unconciousness until the following morning. On the third day a left-sided homonymous hemianopsia developed, and then a left hemiplegia. The bladder was temporarily paralyzed. The hemianopsia disappeared on the tenth day, and the hemiplegia gradually got better, with the exception of the lower part of the leg. The patient was lame but could walk. At the end of the seventh week Eulenburg found the lower face muscles weaker on the left side than on the right, and also the left arm weaker than the right. The gait was faltering, and the left leg stiff. Movements of the foot and lower leg were only just possible. Sensation was considerably affected. There was pain in the back of the head. Progressive improvement ensued. Eulenburg thought that the bullet had penetrated to the right of the sella turcica, injuring the right optic tract and the right crus. By radiography Buka showed that the ball was in the middle fossa of the skull to the right of the middle line.

CASE II. A man, aged thirty-three, attempted suicide ten years ago. The revolver wound was in the hinder part of the right temporal region. At first there were signs of intracranial pressure. For the first four years only slight symptoms were noted. Attacks of pain in the head supervened, and the patient thought that the bullet must still be present. He was then in an asylum for five years, and discharged as incurable. After his discharge he seems to have been able to work. When seen by Eulenburg the man was pale and wasted. Hardly a trace of bodily or mental symptoms could be made out. There was occasional headache in the right supraorbital and temporal regions. There was thus no clinical evidence that the bullet was still present. It was, however, shown by Buka, by means of the Roentgen rays, that the bullet was situated in the middle fossa of the skull behind the right orbital fissure—Brissaud and Londe (*Sem. Med.*, June 24, 1886,) report a case in which a man had been struck by a revolver bullet (calibre 7 mm.) in the middle of the left frontal eminence on August 4, 1895. Various symptoms followed, and finally a spasmodic left hemiplegia of both limbs and face remained. The upper fibers of the facial, the motor oculi, and the masseteric nerves were not involved. The Roentgen rays showed the outline of the skull, the frontal eminence and sinus, the maxillary sinus, petrous and malar bones, zygoma, orbital cavity, etc. The bullet was seen situated in the posterior region at the level of the second temporal convolution, probably above the tentorium. This position corresponded exactly with the direction of the track deduced by the resulting paralyses. The chief interest, besides the exact localization of the missile, consists in the fact that the bullet, being in the temporal region, could not be the cause of the hemiplegia. The latter was due to the section of fibers met with by the bullet—that is it had a capsular, not cortical, origin, and therefore could not have been benefited by any operation. An exposure of an hour and three-quarters was given, and the image would have been still clearer but for a slight clonic movement of the head.—*American Practitioner and News.*

